# Collagen organization of renal cell carcinoma differs between low and high grade tumors

**DOI:** 10.1186/s12885-019-5708-z

**Published:** 2019-05-23

**Authors:** Sara L. Best, Yuming Liu, Adib Keikhosravi, Cole R. Drifka, Kaitlin M. Woo, Guneet S. Mehta, Marie Altwegg, Terra N. Thimm, Matthew Houlihan, Jeremy S. Bredfeldt, E. Jason Abel, Wei Huang, Kevin W. Eliceiri

**Affiliations:** 1 Department of Urology, University of Wisconsin-Madison, Madison, Wisconsin USA; 2 Laboratory for Optical and Computational Instrumentation, University of Wisconsin-Madison, 1675 Observatory Drive, Madison, Wisconsin 53706 USA; 3 Morgridge Institute for Research, Madison, Wisconsin USA; 4 Department of Biostatistics and Medical Informatics, University of Wisconsin-Madison, Madison, Wisconsin USA; 5 Department of Pathology and Laboratory Medicine, University of Wisconsin-Madison, Madison, Wisconsin USA

**Keywords:** Collagen organization, Renal cell carcinoma, Tumor grading, Second harmonic generation imaging, Tissue microarray

## Abstract

**Background:**

The traditional pathologic grading for human renal cell carcinoma (RCC) has low concordance between biopsy and surgical specimen. There is a need to investigate adjunctive pathology technique that does not rely on the nuclear morphology that defines the traditional grading. Changes in collagen organization in the extracellular matrix have been linked to prognosis or grade in breast, ovarian, and pancreatic cancers, but collagen organization has never been correlated with RCC grade. In this study, we used Second Harmonic Generation (SHG) based imaging to quantify possible differences in collagen organization between high and low grades of human RCC.

**Methods:**

A tissue microarray (TMA) was constructed from RCC tumor specimens. Each TMA core represents an individual patient. A 5 μm section from the TMA tissue was stained with standard hematoxylin and eosin (H&E). Bright field images of the H&E stained TMA were used to annotate representative RCC regions. In this study, 70 grade 1 cores and 51 grade 4 cores were imaged on a custom-built forward SHG microscope, and images were analyzed using established software tools to automatically extract and quantify collagen fibers for alignment and density assessment. A linear mixed-effects model with random intercepts to account for the within-patient correlation was created to compare grade 1 vs. grade 4 measurements and the statistical tests were two-sided.

**Results:**

Both collagen density and alignment differed significantly between RCC grade 1 and RCC grade 4. Specifically, collagen fiber density was greater in grade 4 than in grade 1 RCC (*p* < 0.001). Collagen fibers were also more aligned in grade 4 compared to grade 1 (*p* < 0.001).

**Conclusions:**

Collagen density and alignment were shown to be significantly higher in RCC grade 4 vs. grade 1. This technique of biopsy sampling by SHG could complement classical tumor grading approaches. Furthermore it might allow biopsies to be more clinically relevant by informing diagnostics. Future studies are required to investigate the functional role of collagen organization in RCC.

## Background

The incidence of renal cell carcinoma (RCC) has been rising, largely due to the incidental detection of asymptomatic kidney tumors on cross-sectional imaging studies such as computerized tomography (CT) scans [1, 2]. Many of these tumors are small and behave in an indolent fashion, regardless of a malignant histology. Additionally, renal tumors are often more prevalent in elderly populations or patients with comorbidities that make surgical extirpation less desirable.

These clinical concerns have led to increased interest in using renal mass biopsy (RMB) to identify lesions of different metastatic potentials in an effort to tailor treatment to the individual patient. While RMB is accurate for distinguishing malignant from benign masses, biopsy may frequently misestimate tumor grade [3–8]. This inaccuracy is problematic because RCC grade is a strong predictor of tumor behavior and whether patients will develop metastatic disease [9]. Improving the ability to accurately assign tumor grade from RMB samples could greatly help counsel patients about the risks and benefits of treatment.

The pathologic grading of many cancers, including renal, is traditionally based on features of cytological atypia of epithelial cells. Evidence in other cancers such as breast has shown that the tumor stroma also plays a vital role in tumor progression [10, 11]. As such, it follows that stromal features may also be used as biomarkers for tumor aggressiveness. In particular, collagen deposition and rearrangement in the stroma have been linked to non-renal cancer progression with cancer cells being shown to migrate along collagen “scaffolds” in vivo [12]. While the collagen patterns in RCC have not been well described to date, optical detection of collagen changes has been demonstrated in a number of cancers including ovarian, breast, pancreatic, and esophageal and linked to important clinical parameters [10, 11, 13–19].

While collagen and other stromal features can be identified with a variety of stains and standard white light microscopy, these techniques often lack quantitative measures, require special stains, and typically limit interpretation to “subjective” analysis by a pathologist. More recently applied advanced microscopy techniques, however, harness the native physical properties of the tissues to generate high-resolution images, suitable for quantitative image analysis. One such optical technique, Second Harmonic Generation (SHG) [14], is label free, provides optical sectioning, high signal to noise data, and is specific to fibrillar collagen. SHG has been used to study a wide spectrum of diseases accompanied by fibrosis, ranging from different kinds of cancer to atherosclerosis, by providing quantitative information about collagen changes [10–13, 15, 16, 20–29]. While there has been previous application of SHG to distinguish benign and malignant renal tumors [30], SHG based assessment of collagen organization in different grades of RCC has not been previously reported. To that end, we set out to characterize and quantify collagen changes in high and low grade RCC tissues that have different nuclear features but have never been compared in terms of collagen fiber based features.

To summarize, as the traditional pathologic grading for human RCC has low concordance between biopsy and surgical specimen [3–8], there is a need to investigate adjunctive pathology technique that does not rely on the nuclear morphology that defines the traditional grading. Changes in collagen organization in the extracellular matrix have been linked to prognosis or grade in breast [10], ovarian [13, 19], and pancreatic [15] cancers, but collagen organization has never been correlated with RCC grade. In this study, we used SHG based imaging to quantify possible differences in collagen organization between high and low grades of human RCC.

## Methods

### Human RCC microarray slide preparation

A human RCC tissue microarray (TMA) block was constructed by the Translational Research Initiatives in Pathology lab at the University of Wisconsin-Madison (UW-Madison). A section of 5um thickness was cut from the TMA block containing ~600um diameter tissue TMA cores. The spots (cores) were taken from surgical resection (partial or total nephrectomy) specimens. Each core was taken from its representative tumor grade area. Since each core is rather small (0.6 mm in diameter), the tumor grade of each core was homogenous. The section was then placed on a glass slide, stained with standard hematoxylin and eosin (H&E), and mounted under a #1.5 glass coverslip. Different tissue cores were from different patients.

### Histological imaging and region of interest (ROI) annotation

A bright field image of the entire H&E slide was collected with an Aperio CS2 Digital Pathology Scanner (Leica Biosystems) at 20× magnification. Each core of grade 1 and grade 4 was cropped to the size of 1520 pixels by 1520 pixels using Aperio ImageScope viewing software (Leica Biosystems). An expert in genitourinary pathology reviewed the cropped cores to re-confirm the grade information and selected the representative regions containing cancer cells and an adjacent stroma region with patterns consistent with Fuhrman grade the whole tumor had been categorized as. For each core, 2–3 ROIs with the size of 400 pixels by 400 pixels (202um by 202um) were annotated containing typical cancer cells of grade 1 or grade 4 while excluding confounding tissue features (i.e. adipose tissue, benign tissue). In total, 75 TMA cores were verified and annotated as grade 1 clear cell RCC and 55 TMA cores as grade 4 clear cell RCC. Each core represents an individual patient. As described below in the SHG imaging methods section, nine total cores were excluded for lack of signal.

### SHG imaging

All cores in this study were imaged with a custom built forward detection SHG microscope utilized previously [31, 32]. A MIRA 900 Ti: Sapphire laser (Coherent, Santa Clara, CA) was used to deliver 780 nm light to the sample using a 40×/1.25NA water immersion objective (Nikon, Melville, NY). Forward channel light was collected using a 1.2 NA condenser (Nikon, Melville, NY) and the SHG signal was filtered with a bandpass filter specific for the collagen signal at 780 nm (390/18 BP, Semrock) and collected with a H7422–40P GaAsP photomultiplier tube (Hamamatsu, Hamamatsu, Japan). Circular polarization was implemented and verified for the SHG light source. All of the cores were imaged with consistent power settings as a montage of 1024 pixel by 1024 pixel image tiles using in house developed acquisition software (http://loci.wisc.edu/software/wiscscan). No SHG signal was observed for five grade 1 cores and four grade 4 cores after navigating the system to at least 3 different fields of view on each core. Hence those 9 cores were excluded. Seventy grade 1 cores and 51 grade 4 cores with SHG signal were imaged and analyzed in this study.

### Image and statistical analysis

The image tiles of each core were stitched according to image metadata to create a whole core image using Fiji [33]. To locate the annotated ROIs on the SHG images, the corresponding H&E bright field images of each core were first registered with the SHG images using a combined method of color based segmentation via K-means clustering and iterative intensity-based registration implemented in MATLAB (R2014b, The MathWorks Inc., Natick, MA). The ROIs might present a minor shift from the original ROI region during the image registration and ROI transformation. The automatic registration failed in one core due to the weak collagen signal in that core, and that core was manually registered using a Fiji plugin “landmark correspondences”. The pathologist confirmed that the registration process did not alter any clinically relevant image features.

Cropped SHG images were then analyzed using an open-source fiber analysis software tool named CT-FIRE (https://loci.wisc.edu/software/ctfire) [34] as well as other MATLAB (R2014b, The MathWorks Inc., Natick, MA) scripts and R [35] scripts. Collagen density and collagen alignment were calculated for each core and compared between grade 1 and grade 4 based on collagen fibers extracted by CT-FIRE. Collagen fibers that were longer than 5.3 μm (or 30 pixels) were counted as valid fibers in this study. This 5.3u threshold was determined by a qualitative assessment. Collagen density was calculated as the number of fibers in each ROI. Collagen alignment was a measure of the similarity of the orientations of collagen fibers in a given image, calculated as the mean resultant vector length in circular statistics [36]. The alignment coefficient ranged from 0.0 to 1.0, where larger alignment coefficients indicate fibers in a given image are more aligned. For this fiber based collagen alignment analysis, the number of fibers in an ROI was found to need at least 20 fibers to yield statistically reasonable results. This requirement led to the exclusion of some of the cores with very few collagen fibers. Hence, in collagen alignment analysis, the sample size was reduced and was smaller than that used in collagen density analysis.

Summary statistics were calculated for measurements across all ROIs. To compare grade 1 vs. grade 4 measurements, a separate linear mixed-effects model with random intercepts to account for the within-patient correlation (due to multiple measurements from different ROIs per patient) was created for the dependent variables of collagen density and collagen alignment. Each collagen feature was the outcome of interest, and grade 4 vs. grade 1 was the predictor variable. Statistical tests were two-sided, and the level of significance was set at (*p* < 0.05). All statistical analysis was done in R 3.3.1 [35] including the “nlme” package.

## Results

**Fig. 1:**
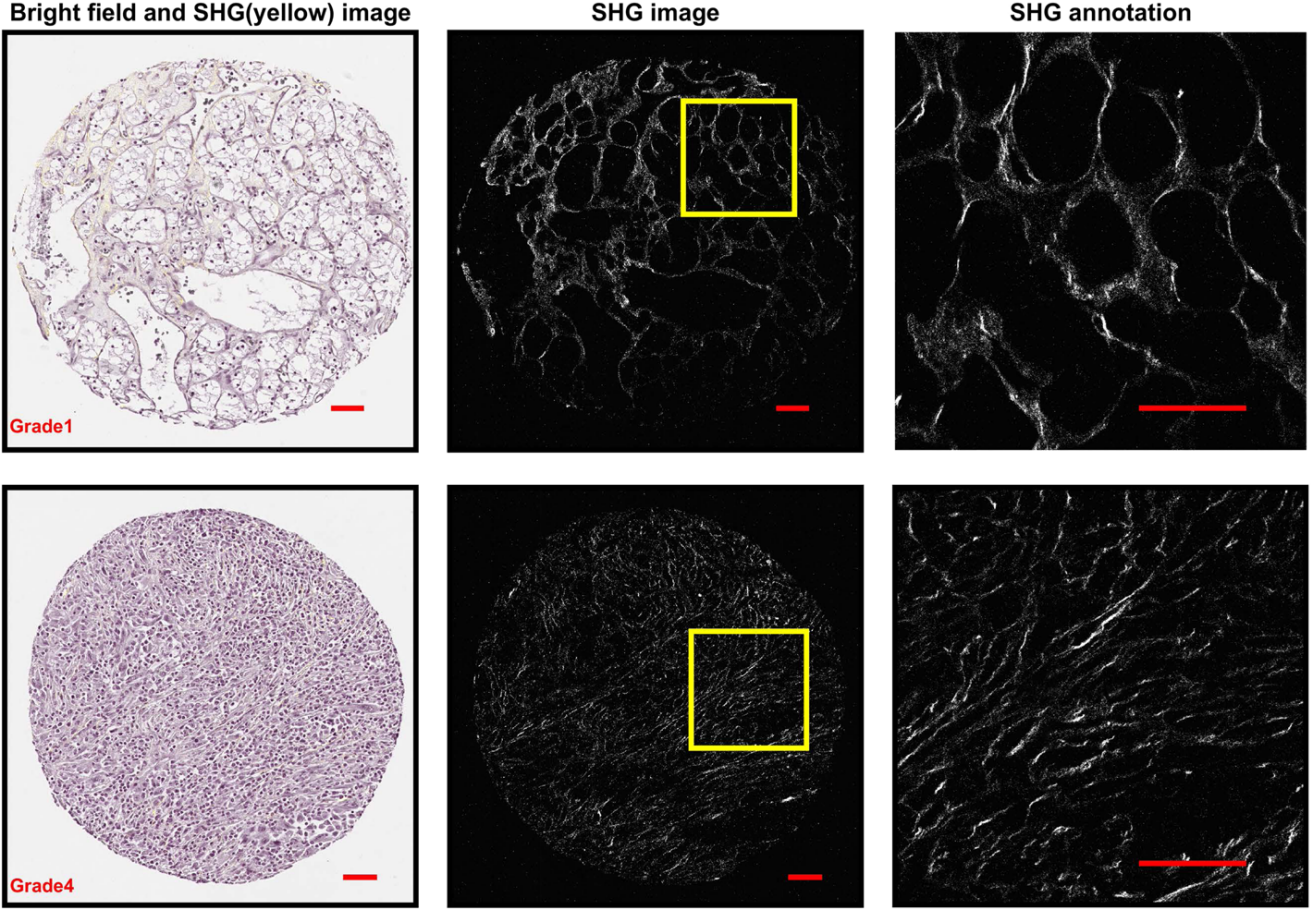
Bright field image and SHG images of two representative cores of RCC grade 1 and grade 4. The fibers in the zoomed-in region of grade 1 have a mesh-like organization, while those in the grade 4 are more aligned. Image brightness and contrast were adjusted for enhanced visualization using MATLAB. Scale bar = 50 um

The Curvelet transform-based image analysis software utilized (CT-FIRE) is capable of quantifying a number of collagen fiber characteristics. CT-FIRE accurately quantified RCC collagen fiber alignment and density based on manual review of CT-FIRE output images with the extracted fibers overlaid on the original SHG image. For this investigation, we focused our analysis on collagen density and alignment, since these were the most apparent differences noted between specimens on subjective assessment. Figure 1 shows two representative bright field and SHG images from grade 1 and grade 4, respectively, that indicate RCC grade 1 and RCC grade 4 have significantly different collagen alignment.

**Fig. 2:**
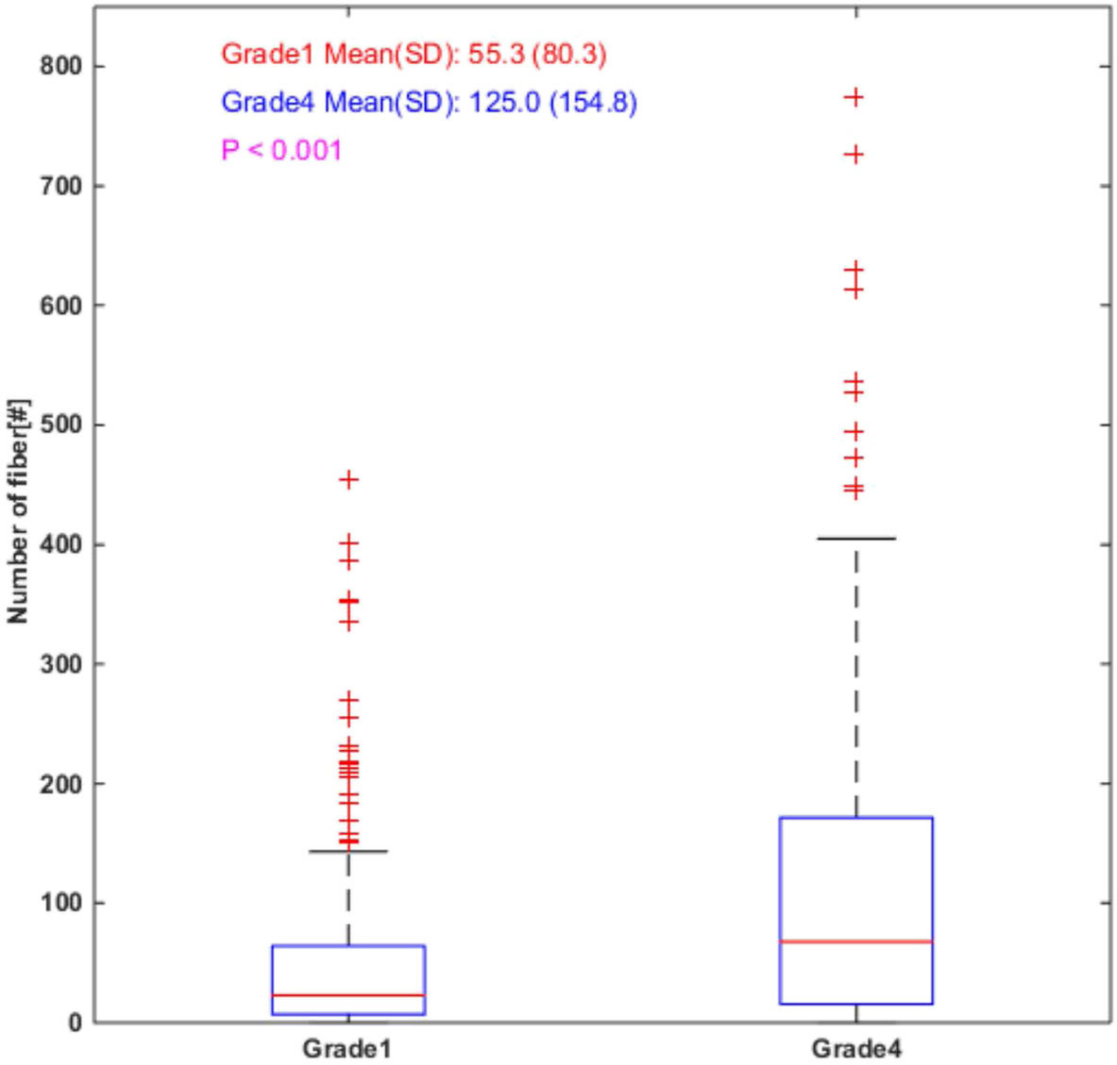
The number of fibers is significantly larger in grade 4 than in grade 1. All 70 grade 1 patients had measurements for 3 ROI for a total of 210 observations, while the 51 grade 4 patients had this data available for 2–3 ROI, for a total of 152 observations. On each box, the central red line represents the median, the lower blue line and the upper blue line represent the 25th and 75th percentiles, respectively, the dashed lines and black lines indicate the lower and upper limits of the data points that are not considered as outliers, and the red crosses represent outliers. The boxplot was drawn using the standard boxplot function with default settings in MATLAB R2014b

Collagen density was found to be significantly lower in grade 1 RCC compared to grade 4 (*p* < 0.001) as shown in Fig. 2, with a density increase of 68.98 for grade 4 RCC (95% confidence interval 30.76–107.20).

**Fig. 3:**
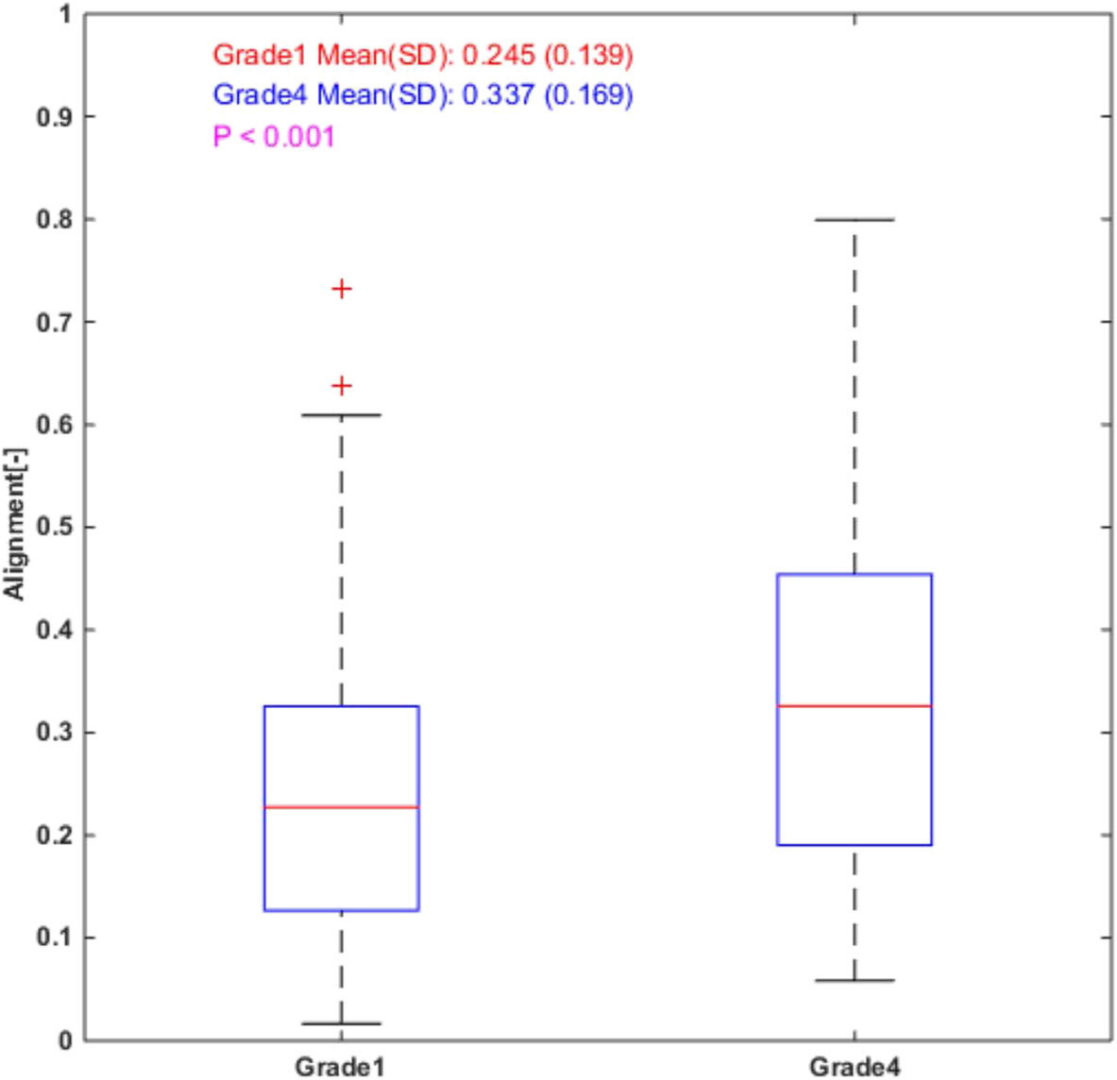
Fiber alignment coefficient is significantly higher in grade 4 than in grade 1. In the alignment comparison, the sample size is smaller than that in collagen density comparison due to the minimum fiber number requirement in the alignment calculation described in section 2.3. 53 patients out of 70 grade 1 patients had alignment measurements for 1–3 ROI for a total of 115 observations, while 43 out of 51 grade4 patients had this data available for 1–3 ROI for a total of 110 observations. On each box, the central red line represents the median, the lower blue line and the upper blue line represent the 25th and 75th percentiles, respectively, the dashed lines and black lines indicate the lower and upper limits of the data points that are not considered as outliers, and the red crosses represent outliers. The boxplot was drawn using the standard boxplot function with default settings in MATLAB R2014b

Significant differences in collagen alignment were also found between grade 1 RCC and grade 4 RCC (*p* < 0.001) as shown in Fig. 3. Collagen fibers appeared more aligned in RCC grade 4 compared to RCC grade 1, with an increase of 0.092 (95% confidence interval0.044–0.141). Parallel “bundles” of collagen fibers were detected in many samples of RCC grade 4.

## Discussion

The increased incidence of small renal masses (SRMs) has placed a significant burden upon patients and the medical community as a whole as clinicians struggle to predict which patients would benefit from treatment of their lesions. It is already accepted that not all patients require surgical extirpation of their renal tumors, but the pathways to making that decision are currently ill defined. Tumor size and growth patterns have been advocated as possible metrics, but low metastatic-potential lesions may be relatively large at diagnosis and a number of studies have demonstrated tumor growth kinetics do not reliably differentiate benign and malignant lesions [37, 38].

Many experts have promoted utilizing RMB to assist in risk stratification [4, 6]. Percutaneous tumor biopsy is appealing due to its low morbidity [4, 39, 40]. While core biopsy can be very useful in separating benign from malignant renal masses, the biopsy accuracy for tumor grade is unfortunately limited. For example, Neuzillet and colleagues [3] found core biopsies to be only 69.8% accurate in determining Fuhrman grade when biopsy pathology reports were compared to surgical specimens. Additionally, Halverson et al. [6] reported that 32 out of 72 patients diagnosed with low-grade clear cell RCC on biopsy who ultimately underwent surgical tumor removal were actually found to have high-grade RCC on final pathology, suggesting an unmet need to predict tumor aggressiveness from small biopsy specimens with increased accuracy. The concordance can be increased if two-tier grading system (e.g. low grades (1,2) vs grades (3, 4)) is used as reported by Millet et al. [5]. However, the level of improvement is case-specific and it remains challenging in more accurate grading between individual grades in low and high grades such as differentiating grade 2 and 3 that is clinically important. This inaccuracy in grading is likely multifactorial and related to challenges in Fuhrman grading, interobserver variations, and most importantly, the tumor grade heterogeneity associated with RCC itself [4].

While collagen characteristics of RCC are not well described, collagen patterning has been linked to tumor behavior and clinical factors in other malignancies. SHG microscopy has already shown promise in studying other cancers, including breast [10], ovarian [13, 19], pancreas [15], and prostate [41–43]. For example, SHG data have been used to differentiate different types of benign and malignant tissues [14, 15]. Keely and colleagues [11, 44] have done extensive work characterizing the collagen patterning associated with breast cancer using SHG, showing that collagen fibers become aligned perpendicular to the tumor boundary in the invasive phase of disease. This “tumor-associated collagen signature” (TACS-3) can serve as an optical biomarker to independently predict breast cancer patient survival [10, 31].

Our current study determined that there are significant differences in both collagen density and fiber alignment between grade 1 and grade 4 RCC. Collagen density comparison from fiber-based analysis shown in Fig. 2 is in agreement with collagen intensity based analysis (quantitation of white pixel percentage to total pixel number), indicating number of collagen fibers and collagen percentage carry similar density-related information in this study. It is particularly compelling that collagen fibers appeared more aligned in RCC grade 4 compared to RCC grade 1 (*p* < 0.001). The prevalence of parallel collagen fibers in high grade RCC is interesting since in other non-renal malignancies, tumor cells have been observed to invade surrounding tissues along aligned collagen fibers [11, 12, 45]. Breast cancer cells have even demonstrated the ability to reorganize collagen fibers in a random collagen matrix to facilitate invasion [11]. The ability of malignancies to organize a collagen “scaffold” may represent an important step in tumorigenesis, and while this active process has not yet been investigated in kidney cancer, it is fascinating to find the greatest collagen alignment in the most aggressive of RCCs. However, the current two collagen features cannot be directly used as diagnostic tools as both features if used alone lack the applicable discriminant ability. It is our hope that future investigations of advanced imaging methods such as SHG, which does not rely on the nuclear morphology that defines the Fuhrman system, may show that this adjunctive pathology technology can improve our characterization of predicted tumor behavior. More studies are needed to determine if/how collagen features could be of help for clinical decision making.

It should be noted that we qualitatively observed that the collagen organization appears to be highly heterogeneous across different cores of a same grade and across different regions of the same core. This observation led us to consult with an expert in genitourinary pathology for specific ROI annotation. Manual ROI annotation improves the accuracy of the grading assignment and makes the comparison between different grades focus on the prominent extracellular matrix features of the RCC tumor at a given grade. Benign tissue was not included in this study as benign tissue is already reliably identified with current techniques and there has already been at least one publication showing the SHG based collagen differences between benign and malignant renal tumor [30].

Our investigation has several limitations that need to be addressed in future studies. First, the sample size is relatively limited and focused on determining differences in collagen patterning between the “extremes” of kidney cancer, low and high grade RCC. Future studies will evaluate the SHG signals generated by grade 2 and 3 clear cell, as well as other renal neoplasms such as papillary RCC and other RCC subtypes. Additionally, RCC is known for its intratumor heterogeneity, which contributes to nuclear grade inaccuracy on RMB specimens and we also observed great heterogeneity of collagen distribution in TMA cores of the same Fuhrman grade. Some cores present very weak collagen signal while many others have strong signals. Whether collagen patterns are more homogeneous throughout a tumor than nuclear features (and thus less sensitive to sampling error) remains yet to be determined. One focus of the future investigations would be sampling larger tumor areas with different Fuhrman grades of the same patient to access how collagen features can possibly help overcome the heterogeneity issue. Finally, as the samples in the microarray were randomly oriented on the slide, we were not able to assess collagen alignment with respect to the tumor margin with the surrounding normal kidney. To quantify the relative alignment, a bigger area of a tissue section rather than just TMAs needs to be imaged and the tumor boundaries could then be annotated and analyzed following the work flow described in our previous work [31].

## Conclusions

While differences in collagen patterning can be visualized subjectively, an advantage of our methodology is that the use of SHG to identify collagen fibers in a sample permits automatic digital image processing and quantification and can be applied to unstained tissues including fresh tissue. From a clinical standpoint, SHG is also a highly flexible platform, able to image unstained tissue (fresh, frozen, or fixed) slides already produced as part of the standard pathology workflow. Additionally, the ability to study tissues with SHG in vivo including in live animal models, such has been previously described in breast cancer, may shed significant light on the functional role of collagen in relation to the behavior of RCC tumors. Finally, despite the limited number of the TMA cores in our investigation, we found significant differences in collagen alignment and density. This is important clinically, due to the nature of the current “problem” facing physicians counseling RCC patients before treatment: a need for more reliable biopsy-based biomarkers with which to advise patients about the malignant potential of their tumors. Future studies will seek to assess if SHG optical biomarkers of collagen alignment and density can improve accurate determination of RCC tumor grade from biopsy specimens and aid patient counseling about cancer prognosis.

## Abbreviations

CT: computerized tomography
H&E: hematoxylin and eosin
RCC: human renal cell carcinoma
RMB: renal mass biopsy
ROI: Region of Interest
SHG: Second Harmonic Generation
TACS: tumor-associated collagen signature
TMA: tissue microarray
UW: University of Wisconsin
